# Comparison of Mean Glandular Dose between Full-Field Digital Mammography and Digital Breast Tomosynthesis

**DOI:** 10.3390/healthcare9121758

**Published:** 2021-12-19

**Authors:** Kar Choon Teoh, Hanani Abdul Manan, Norhashimah Mohd Norsuddin, Iqbal Hussain Rizuana

**Affiliations:** 1 Department of Radiology, University Kebangsaan Malaysia Medical Centre, Jalan Yaacob Latif, Bandar Tun Razak, Cheras 56000, Malaysia; kcteoh.87@gmail.com; 2 Makmal Pemprosesan Imej Kefungsian (Functional Image Processing Laboratory), Department of Radiology, University Kebangsaan Malaysia Medical Centre, Jalan Yaacob Latif, Bandar Tun Razak, Cheras 56000, Malaysia; hanani@ukm.edu.my; 3 Diagnostic Imaging & Radiotherapy Program, University Kebangsaan Malaysia, Kuala Lumpur 50586, Malaysia; norhashimahnorsuddin@ukm.edu.my

**Keywords:** mean glandular dose, mammography, breast cancer and digital breast tomography

## Abstract

Early detection of breast cancer is diagnosed using mammography, the gold standard in breast screening. However, its increased use also provokes radiation-induced breast malignancy. Thus, monitoring and regulating the mean glandular dose (MGD) is essential. The purpose of this study was to determine MGD for full-field digital mammography (FFDM) and digital breast tomosynthesis (DBT) in the radiology department of a single centre. We also analysed the exposure factors as a function of breast thickness. A total of 436 patients underwent both FFDM and DBT. MGD was auto calculated by the mammographic machine for each projection. Patients’ data included compressed breast thickness (CBT), peak kilovoltage (kVp), milliampere-seconds (mAs) and MGD (mGy). Result analysis showed that there is a significant difference in MGD between the two systems, namely FFDM and DBT. However, the MGD values in our centre were comparable to other centres, as well as the European guideline (<2.5 mGy) for a standard breast. Although DBT improves the clinical outcome and quality of diagnosis, the risk of radiation-induced carcinogenesis should not be neglected. Regular quality control testing on mammography equipment must be performed for dose monitoring in women following a screening mammography in the future.

## 1. Introduction

### 1.1. Background of Mammography

Since the late 1920s, mammography has been the gold standard for early detection of breast cancer [1,2]. It helps reduce the mortality rate up to 30%, and the remission rate too [3]. Therefore, an annual mammogram is necessary for women, especially after the age of 45 [4]. The number of new cases in Malaysia reported by the International Agency for Research on Cancer in 2020 [5] was 8418 per 100,000 individuals (32%). Breast cancer is the most common cancer among women.

Initially, early breast cancer imaging was conducted by conventional two-dimensional (2D) screen-film mammography (SFM) in the craniocaudal (CC) view and the mediolateral oblique (MLO) view [1,2,6]. In January 2000, full-field digital mammography (FFDM) replaced SFM. FFDM uses digital images: X-rays are converted into electrical signals, and are processed and displayed as digital imaging on a computer screen [6]. The use of digital technology enables the manipulation of images to improve contrast and resolution [6]. Compared to SFM, FFDM has demonstrated significantly higher breast cancer detection rates (0.59% for FFDM and 0.38% for SFM (*p* = 0.02)) [6]. However, a study showed there were no significant differences between FFDM and SFM in terms of missed breast cancer (10.5% for FFDM and 8.1% for SFM (*p* = 0.77)) [7]. New advanced imaging techniques, such as breast tomosynthesis and contrast-enhanced digital mammography, are used to improve the sensitivity of breast cancer detection [6].

Digital breast tomosynthesis (DBT) uses X-rays and computer reconstruction to create three-dimensional (3D) images. Zuckerman et al. [8] demonstrated that DBT screening significantly increases the sensitivity of breast cancer detection and reduces the recall rates. However, the average glandular dose per exposure for DBT was considerably higher than that for FFDM or SFM, at approximately 34% above the average [9]. A C-view software or synthesised 2D (s2D) image was developed, where the 2D images are created by collapsing the 3D tomosynthesis images into a single slice through the process of frequency-weighted reconstruction. This is the same principle as maximum intensity projection reconstruction in magnetic resonance imaging and computed tomography. The radiation dose is reduced, as the patient is exposed to DBT imaging only once.

### 1.2. Radiation Dose and the Estimated Risk in Mammography

Multiple mammographic views in the case of large breasts are often required for diagnostic purposes. This causes patients to have a greater risk of radiation-induced breast cancer [10]. Thus, screening age and frequency significantly affect the radiation-induced breast cancer incidence rate and mortality [11].

Awareness of the risk of radiation-induced breast carcinoma is higher nowadays. Radiation dose evaluation and risk assessment are necessary to outweigh the risks against benefits before performing the procedure [12]. Therefore, to reduce radiation exposure, optimisation of the procedure and radiation dose is required.

The radiation dose of mammography is estimated by calculating the mean glandular dose (MGD) [13]. MGD is the most appropriate dosimetry quantity to predict radiation-induced carcinogenesis risk in mammographic practice [13]. There are two principal methods to assess mammography-related MGD: a standard breast phantom and a patient-based measurement. They both define MGD limits and are well-suited for quality control and inter-system comparisons, to ensure that all units achieve acceptable doses. Such measurements, however, do not indicate the dose received by the patient [2,3] After each exposure, modern FFDM units display the MGD value and the entrance or incident air kerma (K) to the breast. Information on the calculation of these values is limited, and knowing how MGD values compare to and correlate with conventional Monte Carlo-based methods is useful [4]. 

Entrance surface air kerma (ESAK) and half-value layer (HVL) indirectly estimate MGD. It has been shown that ESAK and conversion coefficient based on Monte Carlo calculations extrapolate MGD for standard breast projections. The mean ESAK and MGD were 4.4 ± 1.1 mGy and 1.1 ± 0.3 mGy, respectively. Radiation-induced cancer due to mammography was estimated to be 177 × [10]^6^, with a statistically significant relationship (*p* < 0.01) between MGD, tube voltage product (mAs) and breast thickness (mm). The breast cancer risk is increased significantly if there is repeated radiation exposure [14]. Therefore, it is essential to lower the radiation dose to the patient, to reduce the risk of radiation-induced breast cancer [14].

### 1.3. Practice of Mammographic Techniques in Hospital Canselor Tuanku Muhriz (HCTM)

Hologic Selenia Dimensions mammography has been used in HCTM since August 2019. In HCTM, the combination technique, also known as combo technique, was initially used, in which FFDM imaging was combined with 3D breast tomosynthesis or DBT. In the same setting, s2D imaging was also created from 3D data. The reason for using the combo technique was for the radiologists to get accustomed to the new mammographic approach. Currently, in HCTM, instead of using the combo technique for mammography, we have switched to 3D breast tomosynthesis, and the s2D images are created from 3D data, overcoming the necessity of acquiring conventional 2D images.

Since the installation of the new mammography technique, MGD in our patients has not been evaluated or quantified for DBT. This study aims to compare MGD between two breast imaging techniques, namely FFDM and DBT in HCTM, as well as MGD with similar studies in other countries. We also aim to analyse the exposure factors as a function of breast thickness.

From our literature search, the present study is the first in our county to publish data comparing FFDM and DBT. We hope the results will be utilised for future DBT guidelines in our country.

## 2. Materials and Methods

### 2.1. Subjects and Procedures 

The digital mammography Hologic Selenia Dimensions machine console was used for both FFDM and DBT breast imaging. An amorphous selenium detector with an active imaging area of 9.2 inches × 11.2 inches (or 23.3 cm × 28.5 cm) was used. The spatial resolution of the image receptor was >0.2 lp/mm in both FFDM and DBT breast imaging. The anode material was tungsten, with two different focal spots of 0.1 mm and 0.3 mm. The automatically selected X-ray beam filtration materials were 0.05 mm rhodium (Rh), 0.05 mm silver (Ag) and 0.7 mm aluminium (Al), corresponding to the exposure factors. Automatic exposure control (AEC) was used to select the appropriate exposure factors (tube voltage in kVp and tube current in mAs) in response to the compressed breast thickness (CBT) for image acquisition based on the manufactured setting. The exposure factor values (tube voltage and tube current), CBT, age, the combination of target/filter and MGD for each image projection were recorded and displayed on Digital Medical Imaging Converter (DICOM) images.

This was a retrospective and prospective study conducted in the Radiology Department of HCTM to evaluate mammographic images taken from August 2019 to May 2020, which is 10 months, using the simple sample randomization method to determine the study sample. A total of 436 patients and 3600 images were analysed. Craniocaudal (CC) and mediolateral oblique (MLO) projections were taken for all FFDM and DBT breast imaging patients. In DBT, the X-ray tube moved in an arch (−20° to +20°), while image acquisition and the resulting images were reconstructed into a series of DICOM images.

### 2.2. Ethical Considerations 

The institution’s research and ethics committee of HCTM approved the study (ethical code: FF-2020-399). This was a retrospective descriptive study where the patient records were made anonymous and de-identified before analysis. Thus, no informed or written consent was required.

### 2.3. MGD

MGD is used in mammography as a dose quantity and is defined as the average dose to the glandular tissue within the breast. It is estimated based on the standard breast parameters ESAK and HVL. There are standard two-step protocols to determine MGD. First, the ESAK to the breast is determined. Then, MGD is determined by multiplying the surface exposure value by published dose factors. The dose factor values are auto-regulated according to the breast thickness. The mean value presented in this study is based on the following equation [15,16,17]:

MGDT = ESAKT × g × c × s

where T is the conversion factor (mGy/R or mrad/R), g is the conversion factor for 50% glandular breast based on the thickness and HVL, c is the correction factor based on non-standard glandular breast/thickness and s is the correction factor based on non-molybdenum anode/filter combination.

In this study, the MGD values were auto calculated by the mammography machine and recorded directly on the system based on the manufacturer’s protocol for all the image projections for both FFDM and DBT. The value was displayed with other variables, such as ‘patient’s age’, ‘compressed breast thickness’, ‘target/filter combination’ and ‘exposure factors (kVp and mAs)’ on the DICOM images.

### 2.4. Sample Size Calculation and Statistical Analysis

Using Statistical Package for Social Sciences (SPSS), a paired t-test was applied to assess the statistical significance of the differences between DBT and FFDM in terms of MGD-related variables. The MGD values from FFDM and DBT were then analysed for breast thicknesses, exposure factors and force compression. A linear regression test was finally performed to test whether there was any correlation between the different mammographic parameters. *p*-values of <0.05 were significant. 

For the sample size calculation, we used Slovin’s formula as below:

$$n=\frac{N}{1+Ne^{2}}$$

where *n* is the sample size, *N* is the population size, *e* is the margin of error (0.04) and 1 is the constant value. Applying this formula, we found that the sample size should be *n* = 152, as shown below:


$$N=\frac{200}{1+\left(200\right)0.04^{2}}=152$$

## 3. Results

### 3.1. Sample Characteristics

Combining retrospective and prospective studies during a period of 10 months, 436 patients were included in this study, with 28 having undergone right mastectomy and 45 patients left mastectomy. 

### 3.2. MGD of FFDM and DBT

The MGD values of FFDM and DBT are presented in Table 1 and Table 2, respectively. MGD of FFDM was in the range of 1.42 to 1.49 mGy in the CC view and 1.74 to 1.8 mGy in the MLO view. As for DBT, MGD fluctuated from 1.84 to 1.90 mGy in the CC view and 2.17 to 2.24 mGy in the MLO view. Comparing FFDM with DBT, there was a significant increase in MGD in DBT for both CC (27.5–29.5%) and MLO views (24.4–24.7%). In all views, the paired sample t-test showed a significant difference in the MGD (*p* = 0.001) between the FFDM and DBT techniques (Table 3).

**Figure 1:**
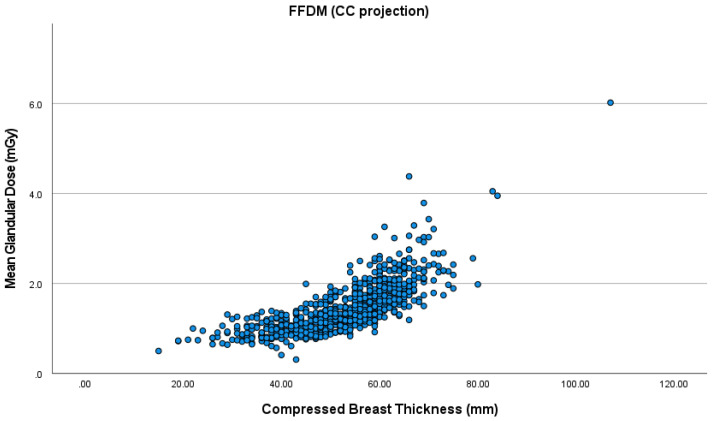
Mean glandular dose (MGD) in full-field digital mammography (FFDM) in the craniocaudal (CC) projection.

**Figure 2:**
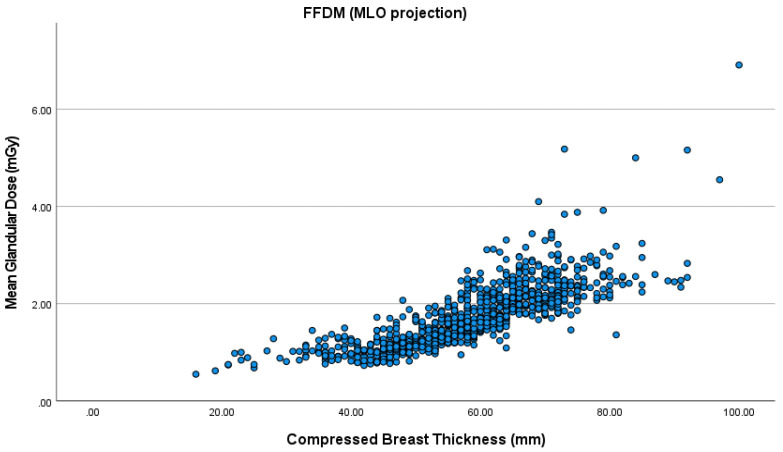
MGD in FFDM in the mediolateral oblique (MLO) projection.

**Figure 3:**
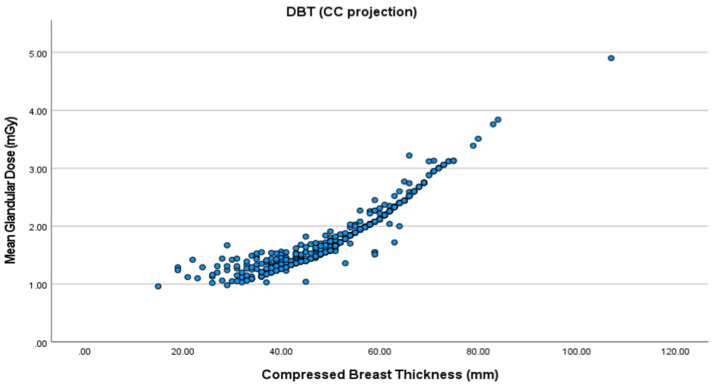
MGD in digital breast tomosynthesis (DBT) in the CC projection.

**Figure 4:**
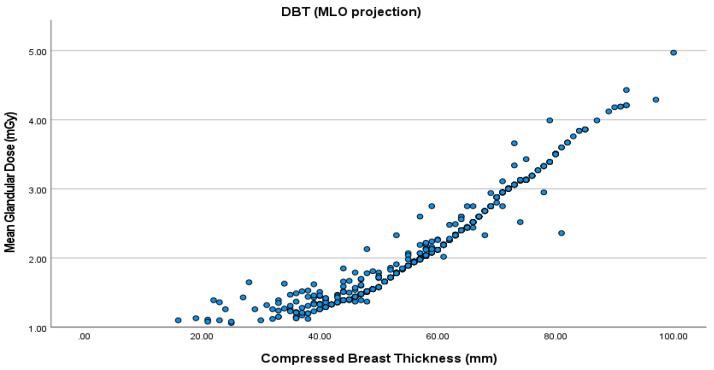
MGD in DBT in the MLO projection.

The mean CBT was 52.4 cm in the right and 53.6 cm in the left CC view. As for the MLO view, the mean CBT was 58.3 cm for the right and 59.4 cm for the left breast. MGD increased with higher CBT for all the views in both FFDM and DBT (Figure 1, Figure 2, Figure 3 and Figure 4). The MLO view had higher MGD values than the CC view, and this is due to higher values of the corresponding exposure factors. The paired sample t-test also showed a significant difference in MGD (*p* = 0.001) in relation to CBT (Table 4).

### 3.3. Comparison with Other Healthcare Centres 

Comparing our results with those from similar studies conducted in other healthcare centres, the value of MGD was in good agreement with the European guidelines in different countries (Table 5).

## 4. Discussion

The gold standard for breast cancer screening and early detection of breast lesions has been FFDM, as it is a cheap, fast and non-invasive technique [1]. However, FFDM causes tissue superimposition and a reduced rate of breast lesion detection [9]. Thus, DBT is a more favourable technique, as it reduces recall rates and improves the detection rate of breast lesions in a dense breast, thus, reducing the rate of false-negative biopsies [9]. However, DBT uses higher radiation doses. In a total of 4780 FFDM and 4798 DBT images from 1208 women enrolled in screening trials to compare the ground dose [9], MGD was calculated based on Dance’s model using processed raw images [18]. DBT and FFDM were compared in terms of AEC and MGD levels. The result showed statistically significant differences in MGD between FFDM and DBT in all projections. In the CC projection during FFDM, MGD was 3.37 mGy, while that of DBT was 1.86 mGy (*p* < 0.001). In the MLO projection during FFDM, MGD was 1.37 mGy, and for DBT it was 1.88 mGy. From these results, it was shown that the radiation dose of DBT is slightly higher compared to FFDM [19]. 

According to Baek et al. [20] two major factors affecting radiation dose in mammography are breast thickness and breast density. The DBT radiation dose was 13% higher than in FFDM (2.32 mGy in DBT versus 2.05 mGy in FFDM) for a breast thickness of 50 mm. However, this difference is smaller when breast thickness exceeds 50 mm [19]. As the breast thickness increases, the dose increases at a slower rate in DBT but aggressively in digital mammography. This is because digital mammography’s tube current exposure time (mAs) is higher than that of DBT [19,20].

Our study showed that doses for CC images were on average 20% lower than those for MLO images in FFDM. As for DBT, the CC images were only 3% lower than those for MLO images. This could be due to the differences in thickness between CC and MLO view. Furthermore, the pectoral muscle may be overlying the cassette detector in the MLO view. As the CC images have less radiation dose than those of the MLO view, additional images such as magnifications or spot compression view are recommended. Biopsies should also be executed on the CC projection whenever possible.

Our study also showed that DBT doses are higher than FFDM by 24.7%. This may be owing to DBT’s cumulative sum dose resulting from the combination of several projection images. The individual images are then reconstructed into a series of images. Our institute has currently replaced FFDM with an s2D view, which is a reconstructed image from DBT acquisition, reducing the radiation dose. Previous studies from other countries support the finding of a significantly lower radiation dose in 2D images [21,22,23,24]. 

Screen-film mammography was used in the studies by Jamal and Chavelier [7]. Hermann et al. [25] found that MGD is reduced approximately to 25% when using a full-field digital detector compared to screen-film mammography. A similar study carried out by Gosch et al. [26] showed that there was a 20% dosage reduction when using a selenium detector or caesium-iodide/amorphous silicon (a-Si) detector.

The usage of AEC is also proven to reduce radiation doses compared to manual exposure control. Studies show that the image quality using AEC is improved due to minimising human errors in determining reasonable exposure factors in the examination [27]. Thus, regular calibration and maintenance of mammography play a significant role in radiation protection. 

Different target/filter combinations alter the patient’s exposure to radiation and the image quality. The combinations of rhodium/rhodium (Rh/Rh), molybdenum/rhodium (Mo/Rh) and molybdenum/molybdenum (Mo/Mo) were used in the studies by Jamal, Chaveliar. M. and W.O. Chijoke [7]. Only S. Saadi used tungsten/silver (W/Ag) as a target/filter in image acquisition. In the present study, tungsten was used as a target. However, the filters were aluminium, rhodium and silver. 

Ulhenbrock et al. [28] showed that using tungsten (W/Rh) as target/filter reduced the dosage by a factor of two compared to Mo/Rh. M. Aminah et al. [29] also discovered a reduction in the dose when using W/Rh, followed by Mo/Rh and Rh/Rh. Due to a higher X-ray spectrum for W/Rh compared to Mo/Rh at the same exposure setting, more photons reach the detector, and fewer photons are absorbed by the breast tissue, thus, increasing the signal-to-noise ratio and reducing the radiation dose. Under the same exposure setting, ESAK would also be lower for tungsten. However, this causes a reduction in the contrast of the images, which is not ideal in the case of analogue screen-film mammography. By adjusting the contrast in a digital image, this issue can be overcome [30].

Nevertheless, reducing individual exposure to radiation doses is important, as breast imaging techniques are closely related to increased breast cancer incidence [31,32]. Even though the risk of radiation-induced breast cancer is low, regular monitoring and regulating the standards of breast imaging are important. Repeated diagnostic exposure is an established cause of a higher risk of breast cancer. In this line, screening mammography is not recommended for younger patients (under 40 years). 

MGD per woman was calculated by summing the individual MGD values (MGD from both breasts) and averaging them over both breasts. The mean MGD of FFDM in the CC projection was 1.46 and in the MLO projection 1.77; as for DBT in the CC projection, it was 1.87 and in the MLO projection 2.21. In conclusion, our results are comparable with previous studies (Jamal et al., 2003; Chevalier M. et al., 2003; S. Saadi et al., 2018 and W.O. Chijoke et al., 2017). Even though Chevalier M. et al. (2003) [23] reported a much bigger sample size, the outcomes are still comparable with our study. This shows that our result is within the acceptable limit range. Therefore, it can be used in the future as a guideline.

### Limitations

Although in our study, MGD was lower compared to other healthcare centres, there are no data on risk reduction of radiation-induced carcinogenesis. Therefore, we plan to conduct the second phase of this study to assess the risk of radiation-induced carcinogenesis and monitor the dose in women following screening mammography in the future. The current data are from one centre only, therefore, we plan to conduct a multi-centre study incorporating data from other centres in Malaysia to extend our results with larger sample sizes. Finally, the literature used in the current study is limited due to a lack of new literature available on this particular topic, especially DBT.

## 5. Conclusions

In conclusion, there is a significant difference in MGD between the two systems, namely FFDM and DBT. However, MGD values acquired in our study for both FFDM and DBT were comparable to other centres. Our MGD was also within the acceptable limit of European guidelines and the recommended dosage (<2.5 mGy) per exposure in a standard breast [32]. We are still referring to the recommended dose of the European guideline, as we do not have a local diagnostic reference level in national mammography screening for DBT. We hope that our research may be of assistance to produce a national/local diagnostic reference level for MGD.

The combined effort of radiographers, radiologists and physicists in practicing the ALARA principle which stands for ‘as low as reasonably achievable’ which is vital to achieving our aim. Regular quality control testing on mammography assists in maintaining dose regulation. Therefore, we would like to advocate that implementing DBT in breast cancer detection significantly improves the patient’s clinical outcome and quality of diagnosis. However, the risk of radiation-induced carcinogenesis and dose monitoring in women during screening mammography needs to be regulated at all times.

## Figures and Tables

**Table 1:** Technical exposure factors for each projection.

Projection	kVp	mAs	Target/Filter	Acquisition Mode
RCC	29.2 ± 1.9 (8–32)	126.8 ± 40.2 (11–263)	W/Al, W/Rh, W/Ag	FFDM
RMLO	30.0 ± 2.7 (20–61)	148.3 ± 43.4 (53–345)		
LCC	29.5 ± 2.7 (27–60)	131.4 ± 45.1 (11–361)		
LMLO	30.0 ± 1.7 (25–36)	150.3 ± 47.3 (2–399)		
RCC	31.2 ± 1.9 (25–36)	58.3 ± 9.9 (36.1–120)		DBT
RMLO	32.4 ± 2.9 (26–60)	63.6 ± 10.8 (37.5–120)		
LCC	31.4 ± 2.1 (25–45)	59.0 ± 9.8 (36.8–120)		
LMLO	32.6 ± 2.7 (26–44)	64.2 ± 11.3 (37–120)		

RCC = right cranio-caudal, RMLO = right mediolateral oblique; Data presented as mean ± standard deviation (minimum–maximum).

**Table 2:** Compressed breast thickness (CBT) and mean glandular dose (MGD) per exposure for each projection.

Projection	CBT (cm)	MGD (mGy)	Acquisition Mode
RCC	52.4 ± 10.2 (15–75)	1.42 ± 0.50 (0.31–3.21)	FFDM
RMLO	58.3 ± 12.2 (19–92)	1.74 ± 0.63 (0.62–5.00)	
LCC	53.6 ± 10.8 (19–107)	1.49 ± 0.61 (0.41–6.02)	
LMLO	59.4 ± 12.9 (16–100)	1.80 ± 0.72 (0.55–6.91)	
RCC	52.4 ± 10.2 (15–75)	1.84 ± 0.45 (0.96–3.13)	DBT
RMLO	58.3 ± 12.2 (19–92)	2.17 ± 0.64 (1.06–4.21)	
LCC	53.6 ± 10.8 (19–107)	1.90 ± 0.51 (0.98–4.90)	
LMLO	59.4 ± 12.9 (16–100)	2.24 ± 0.69 (1.04–4.97)	

RCC = right cranio-caudal, RMLO = right mediolateral oblique; Data presented as mean ± standard deviation (minimum–maximum).

**Table 3:** MGD in FFDM and DBT.

View/Projection	Technique	Median MGD (mGy)	*p*-Value
RCC	FFDM	1.42	<0.005
	DBT	1.84	
LCC	FFDM	1.49	<0.005
	DBT	1.9	
RMLO	FFDM	1.74	<0.005
	DBT	2.17	
LMLO	FFDM	1.8	<0.005
	DBT	2.24	

RCC = right cranio-caudal, RMLO = right mediolateral oblique.

**Table 4:** The compressed breast thickness (CBT) median in the craniocaudal (CC) and mediolateral oblique (MLO) projections.

	CC	MLO	*p*-Value
CBT (cm)	R: 52.4	R: 58.3	<0.005
	L: 53.6	L: 59.4	

R: right breast; L: left breast.

**Table 5:** Comparison with other studies.

Data Source	Number of Patients	Mean CBT (mm)	Mean MGD per Film (FFDM)	Mean MGD per Film (DBT)
Present study	462	CC: 52.9 MLO: 58.8	CC: 1.46 MLO: 1.77	CC: 1.87 MLO: 2.21
Jamal et al., (2003) Malaysia (23)	316	CC: 37 MLO: 45	CC: 1.54 MLO: 1.82	-
Chevalier et al., (2003) Spain (26)	5034	52	CC: 1.8 MLO: 1.95	-
Saadi et al., (2018) Algeria (22)	32	CC: 53.1 MLO: 57.9	CC: 1.8 MLO: 2.03	CC: 2.48 MLO: 2.71
Chijoke et al., (2017) Nigeria (25)	427	51.6	CC: 2.21 MLO: 2.63	-
